# Risk factors, clinical profiles, and fungal etiology of toenail onychomycosis among female traders in a central province of Vietnam

**DOI:** 10.22034/cmm.2026.345480.1710

**Published:** 2026-06-07

**Authors:** Kieu-Anh Thi Tran, Khanh-Linh Thi Duong, Thang Doang Thai, Anh Ngoc Do, Loi Ba Cao, Tran-Anh Le

**Affiliations:** 1 Paediatric Department, Internal Medicine, Department of Postgraduate Education, Vinh Medical University, Vinh, Nghe An, Vietnam; 2 Thai Thuong Hoang Hospital, Vinh, Nghe An, Vietnam; 3 Department of Parasitology, Vietnam Military Medical University, Ha Dong, Ha Noi, Vietnam; 4 Scientific and Training Management Department, National Institute of Malariology, Parasitology and Entomology, Ha Noi, Vietnam; 5 Microbiology and Molecular Biology Department, National Hospital for Tropical Diseases, Hanoi, Vietnam

**Keywords:** Dermatophytes, Non-dermatophyte molds, Risk factors, Toenail onychomycosis, Yeast

## Abstract

**Background and Purpose:**

Hot and humid climate of Vietnam favors fungal infections; however, data on toenail onychomycosis in this region remain limited. In this regard, the present study aimed to investigate risk factors, clinical features, and causative agents of toenail onychomycosis in Vietnam.

**Materials and Methods:**

This cross-sectional study was conducted in Nghe An province, Vietnam, from March 2021 to August 2022. Toenail samples from female traders with suspected onychomycosis underwent direct examination and culture. Isolates were identified using conventional and molecular techniques, including restriction fragment length polymorphism analysis and sequencing of the internal transcribed spacer region.

**Results:**

Based on findings, 45.5% of 224 participants were diagnosed with onychomycosis. The identified risk factors for onychomycosis were water contact (odds ratio (OR) 2.434; 95% confidence interval (CI) 1.098–5.383) and nail grooming (OR: 2.096; 95% CI: 1.002–4.384). Distal and lateral subungual onychomycosis was the most common form (84.3%), followed by superficial white (8.8%) and proximal subungual form (6.9%). Most cases were classified as mild (35.3%) or moderate (40.2%) infections, with an average onychomycosis severity index (OSI) score of 10.25 ± 8.09. Yeasts accounted for 70.6% of cases, and *Candida* was the leading genus (54.9%), followed by *Trichosporon* (13.7%) and *Geotrichum* (1.9%). Most mold-related cases were caused by non-dermatophyte fungi, including *Aspergillus* (16.7%), *Penicillium* (5.9%), *Curvularia* (2.9%), *Fusarium* (2.0%), and *Talaromyces* (1%), while dermatophyte (*Trichophyton*) was identified in only one case (1%). Compared with yeast infections, mold-related cases were more associated with older participants and a higher rate of severe cases (43.3% vs. 16.7%, *p* = 0.010) as well as OSI score (13.6 ± 8.2 vs. 8.8 ± 7.7, *p* = 0.006).

**Conclusion:**

Both behavioral and occupational factors were risks for toenail onychomycosis, which was mostly mild to moderate and associated with yeasts. Non-dermatophyte molds were less frequent but linked to more severe cases. Targeted prevention and tailored treatment strategies are needed.

## Introduction

Onychomycosis is a fungal infection of the nail unit with significant clinical and public health implications. It accounts for nearly 50% of all nail disorders and affects 10% of the world population [1, 2]. Both finger and toenail can be infected, but the latter are more frequently involved, largely due to slower growth rates, reduced vascularity, and greater exposure to trauma and occlusion [3]. The contributory factors for onychomycosis include demographic, occupational, and behavioral factors, which may vary across subpopulations [4-6]. Therefore, identification of risk factors within specific groups is essential for developmen of effective and appropriate preventive strategies.

Clinically, onychomycosis is manifested by nail discoloration, thickening, onycholysis, dystrophy, and pain, which negatively affect the quality of life [7]. Treatment of toenail onychomycosis is needed as the infection rarely resolves spontaneously and may persist for years [8]. Management depends on both disease severity and the causative agents. Topical therapy is often sufficient for milder cases, whereas systemic therapy is required for more extensive infections. Historically, terbinafine has been considered the most effective systemic treatment due to its potent antifungal activity against dermatophytes, the most common causes of onychomycosis [8].

However, evidence from different regions has highlighted an increasing proportion of cases attributable to yeasts and non-dermatophyte molds (NDMs) [9-17]. As a result, the effectiveness of terbinafine has become less predictable since it exhibits variable *in vitro* activity against these organisms [18-21]. For that reason, correct identification of the pathogen is essential to optimize treatment, but empiric therapy is still a common approach, particularly in resource-limited settings [22]. This underscores the need for up-to-date information on the distribution of causative agents across different regions to guide more effective therapeutic strategies.

Vietnam is a Southeast Asian country with natural conditions, including high temperature and humidity, that favor fungal growth. Although cutaneous fungal infections are recognized as prevalent dermatological conditions in the country, there is insufficient information about the characteristics of onychomycosis, especially about the role of non-dermatophytic agents [23, 24]. The present study aimed to determine the risk factors, clinical characteristics, and causative agents of toenail onychomycosis among female traders in Nghe An province, Vietnam. This subgroup was selected since they may be at increased risk of onychomycosis due to their occupational exposures, including frequent wet work, prolonged standing, occlusive footwear, and humid or outdoor environments [6, 25].

## Materials and Methods

The present survey was conducted in some markets in Nghe An, a central province in Vietnam. The inclusion criteria were female traders with nail lesions suggestive of onychomycosis who agreed to participate in the study. After obtaining informed consent, the demographic, behavioral, and clinical information was collected using a structured and validated data collection form.

### Ethical approval

This research forms part of a doctoral thesis in Health Studies and received ethical approval from the Ethics Committee of the Vietnam National Institute of Malariology, Parasitology and Entomology (NIMPE) in March 2019 (Approval No. 303/QD-VSR). Participant recruitment adhered to the principles of the Declaration of Helsinki (1964), and all procedures were carried out in accordance with relevant institutional guidelines and national regulations.

### Fungal analysis

Nail samples were obtained by scraping or clipping from the suspected lesion after being cleaned with 70% alcohol. For direct examination, the samples were treated with 10% potassium hydroxide. For isolation, samples were inoculated onto two slants: Sabouraud dextrose agar (SDA, BioMérieux, France) supplemented with gentamicin (0.02 mg/mL) and BBL Mycosel Agar (Becton, Dickinson and Company, USA). Cultures were incubated at room temperature for up to five weeks and considered negative if no colonies appeared.

All yeast colonies were initially identified by subculturing onto CHROMagar^TM^ *Candida* medium and comparing their morphology and color with the descriptions provided in the guidelines of the manufacturers [26]. Representative strains identified on chromogenic medium, together with all unidentified isolates, were subjected to identification by polymerase chain reaction–restriction fragment length polymorphism (PCR–RFLP) analysis based on amplicon size and restriction patterns. Deoxyribonucleic acid (DNA) was extracted using the QIAamp DNA Mini Kit (Cat. No. 51304; QIAGEN, Hilden, Germany) according to the protocol provided by the manufacturer. The PCR was performed with primers internal transcribed spacer1 (ITS1) (5′-TCC GTA GGT GAA CCT GCG G-3′) and ITS4 (5′-TCC TCC GCT TAT TGA TAT GC-3′) (Integrated DNA Technologies, USA) to amplify the ITS1-5.8S-ITS2 rDNA region. The RFLP analysis was conducted with the *Msp*I restriction enzyme [27].

Mold strains were grouped based on their macro- and microscopic morphological characteristics utilizing adhesive tape mounts that are stained with lactophenol cotton blue.

Species-level confirmation was performed by DNA barcoding of the internal transcribed spacer (ITS) regions. Isolates selected for sequencing included: (i) yeasts representing isolates conclusively identified by morphology and PCR–RFLP, (ii) molds representing isolates with similar morphology, (iii) yeast isolates with inconclusive results, and (iv) molds not assignable to any group. GenBank accession numbers OR438032, OR438022, OR437991, OR437989, OR438017, OR438023, and OR437990 were assigned to some representative sequences from this study.

A diagnosis of onychomycosis caused by dermatophytes or yeasts was made if either direct examination or culture yielded a positive result. A diagnosis of NDM onychomycosis was established only when three criteria were met: a positive finding on direct examination, growth of an NDM in culture, and lack of results after repeated attempts to isolate a dermatophyte through culture [28]. The criteria proposed by Hay and Baran were used to classify the clinical types of onychomycosis: distal and lateral subungual onychomycosis (DLSO), proximal subungual onychomycosis (PSO), and superficial white onychomycosis (SWO) [29]. The Criteria of the Classification System for Onychomycosis Severity, based on the Onychomycosis Severity Index (OSI) scoring system described by Carney et al. (2011), were used to grade lesion severity [30]. Water contact was defined as frequent exposure to water during occupational activities, particularly among traders handling seafood. Toenail grooming was defined as professional or intensive toenail care activities, including pedicures, cosmetic toenail treatments, or the use of sharp instruments for cleaning or shaping the toenails.

### Statistics

Data were analyzed in the SPSS software (version 20.0). Categorical variables were described as frequencies (n) and percentages, and associations were assessed using the chi-squared test. Continuous variables were presented as means and standard deviations (SD), and comparisons were performed using the Student’s t-test. Factors showing significant associations in univariate analysis were subject to multivariable analysis. A two-tailed *p* value of < 0.05 was considered statistically significant.

## Results

In total, 224 individuals aged between 15 and 89 years (average 53.9 ± 10.3) participated in the current study. Among them, 102 participants (45.5%) were diagnosed with onychomycosis. Females with a history of cosmetic nail grooming or seafood handling were at significantly higher risk of developing onychomycosis. No other findings differed significantly between participants with and without onychomycosis (Table 1).

**Table 1:** Demographic characteristics of the participants and risk factors of onychomycosis

Parameters		N	Onycho-mycosis	Univariate analysis		Multivariate analysis	
				*p*	OR (CI 95%)	*p*	OR (CI 95%)
Age (years) (mean ±SD)	Onychomycosis	102	54.6 ± 8.9	0.444			
	No	122	53.5 ± 11.3				
Education	Secondary school or lower	140	61 (43.6)	0.489	0.810 (0.471 – 1.394)		
	Higher education	84	41 (48.8)				
Residence	Mountainous areas	129	60 (46.5)	0.787	0.911 (0.535 – 1.552)		
	Plain, coach areas	95	42 (44.2)				
Water contact	Yes	31	20 (64.5)	0.032	2.461 (1.118 – 5.418)	0.028	2.434 (1.098 – 5.383)
	No	193	82 (42.5)				
Soil contact	Yes	60	27 (45.0)	1.000	0.971 (0.536 – 1.759)		
	No	164	75 (45.7)				
Animal contact	Yes	163	76 (46.6)	0.652	1.176 (0.650 – 2.219)		
	No	61	26 (42.6)				
Nail grooming	Yes	36	22 (61.1)	0.046	2.121 (1.023 – 4.401)	0.049	2.096 (1.002 – 4.384)
	No	188	80 (42.6)				
Wearing closed shoes	Yes	62	34 (54.8)	0.069	1.722 (0.955 – 3.106)		
	No	162	67 (41.4)				

Among the 102 patients with onychomycosis, 54 (52.9%) had bilateral nail involvement and 57 (55.9%) had multiple nails affected. The lesions occurred more frequently on the left side (84 cases, 82.4%) compared to the right side (72 cases, 70.6%). More than three-quarters (77 cases, 75.5%) of the participants had mild or moderate onychomycosis, with an average OSI score of 10.25 ± 8.09 (range 2–35). The most clinical type was DSLO (84.3%). Additionally, five individuals had paronychia, and 2 had interdigital lesions (Table 2). Lesions were restricted to the first, second, or third toenail, with 90.5% involving the first (Table 3).

**Table 2:** Clinical characteristics of toenail onychomycosis (n=102)

Variables			Frequency (%)	
Number of involved toenails		1	45	(44.1)
		2	48	(47.1)
		3	9	(8.8)
Site affected	One side	Right	18	(17.65)
		Left	30	(29.41)
	Both sides		54	(52.94)
Clinical types of onychomycosis		DSLO	86	(84.3)
		SWO	9	(8.8)
		PSO	7	(6.9)
Severity of onychomycosis		Mild	36	(35.3)
		Moderate	41	(40.2)
		Severe	25	(24.5)

DLSO: distal and lateral subungual onychomycosis, PSO: proximal subungual onychomycosis, SWO: superficial white onychomycosis.

**Table 3:** Distribution of involved toenails

Toenail	Left	Right	Total	Percentage
1^st^	84	68	152	90.48
2^nd^	7	4	11	6.55
3^rd^	5	0	5	2.98
4^th^	0	0	0	0
5^th^	0	0	0	0
Total	96 (57.14)	72 (42.86)	168	100

According to Table 4, the majority of cases were caused by yeasts (n=72, 70.6%). Three genera of yeasts were identified, with *Candida* being the predominant agent (77.8% of yeast isolates and 54.9% of the total sample), followed by *Trichosporon* (13.7%) and *Geotrichum* (1.9%). Among *Candida* strains, seven species were identified, and *C. albicans* was the predominant agent (46.1% of the total sample and 83.9% of *Candida* isolates). There were 30 females infected with filamentous fungi, and NDM was responsible for most of the infections (29 out of 30 cases, 96.7%). *Aspergillus* was the most common genus (56.7% of filamentous strains and 16.7% of the total sample), followed by *Penicillium* (6 strains, 5.9% of all cases). Notably, only one dermatophyte strain was discovered in our sample. At the species level, *A. flavus* was the predominant mold, representing 26.7% of filamentous isolates (Table 4).

**Table 4:** Distribution of causative agents (n=102)

	Genus	Species	N	(%)
Yeast	*Candida*	*C. albicans*	47	(46.1)
		*C. tropicalis*	3	(2.9)
		*C. parapsilosis*	1	(1.0)
		*C. guilliermondii*	1	(1.0)
		*C. fermentati*	1	(1.0)
		*C. etchellsii*	2	(1.9)
		*C. cylindracea*	1	(1.0)
	*Trichosporon*	*T. asahii*	11	(10.8)
	*Geotrichum*	*T. japonicum*	3	(2.9)
		*G. candidum*	2	(2.0)
Mold	*Aspergillus*	*A. flavus*	8	(7.8)
		*A. niger*	3	(2.9)
		*A. nomius*	3	(2.9)
		*A. clavatus*	1	(1.0)
		*A. sclerotiorum*	1	(1.0)
		*A. versicolor*	1	(1.0)
	*Penicillium*	*P. citrinum*	4	(3.9)
		*P. oxalicum*	1	(1.0)
		*P. polonicum*	1	(1.0)
	*Curvularia*	*C. lunata*	3	(2.9)
	*Fusarium*	*F. solani*	2	(2.0)
	*Talaromyces*	*T. pinophilus*	1	(1.0)
	*Trichophyton*	*T. interdigitale*	1	(1.0)

Analysis showed that patients with mold-related onychomycosis were older than those with yeast infections. The mean OSI score and the proportion of severe onychomycosis were significantly higher in mold-related cases compared with yeast-related cases. It should also be mentioned that no statistically significant association was found between other clinical variables and the causative agent (Table 5).

**Table 5:** The association between fungal aetiology and clinical variables

	Parameters	Yeast (n=72)	Mold (n=30)	p
>Age (mean ± SD)		53.3 ± 9.3	57.6 ± 7.5	0.026*
Clinical type	DLSO (n, %)	59 (81.9)	27 (90.0)	0.383
	Other (n, %)	13 (18.1)	3 (10.0)	
Severity	Severe (n, %)	12 (16.7)	13 (43.3)	0.010*
	Mild – moderate (n, %)	60 (83.3)	17 (56.7)	
OSI score (mean ± SD)		8.8 ± 7.7	13.6 ± 8.2	0.006*
Affected sides	1 (n, %)	37 (51.4)	11 (36.7)	0.197
	2 (n, %)	35 (48.6)	19 (63.3)	
Number of nails	1 (n, %)	34 (47.2)	11 (36.7)	0.385
	2 (n, %)	38 (52.8)	19 (63.3)	

DLSO: distal and lateral subungual onychomycosis,

* significant with *p*<0.05.

## Discussion

### Risk factors of toenail onychomycosis

In total, 224 female traders from local markets in Nghe And province, a central province of Vietnam, participated in the current study. As summarized in Table 1, nearly half of the participants (45.5%) had onychomycosis, aligning with previous estimates that 50–60% of abnormal toenails are attributable to this condition [31]. Results of the analysis suggested that females with a history of cosmetic nail procedures or seafood handling were at a significantly higher risk of developing onychomycosis. This aligns with previous studies, such as those performed by Toukabri et al. (2017), which reported the association between foot mycoses and pedicure (*p* = 0.006) [6].

Cosmetic nail procedures may increase susceptibility to fungal infections due to microtrauma to the nail plate or periungual skin [32, 33], as well as potential exposure to contaminated instruments [34]. Although the association between seafood handling and toenail onychomycosis has not been specifically documented, Le et al. (2020) found that fungal infections of the hand were more common among seafood workers (26.1%) than residents (2.7%) [35]. This relationship may result from the occupational exposures to wet work, prolonged standing, occlusive footwear, and humid or outdoor environments among those selling seafood [6, 25].

In terms of clinical presentation, the participants exhibited the characteristic features commonly associated with toenail onychomycosis. In the present study cohort, onychomycosis lesions frequently affected both feet, [3] with the hallux being the most commonly involved digit, consistent with previous reports [36, 37]. Similarly, the predominant clinical type was DLSO (84.3%) [3], [4]. It is noteworthy that the majority of onychomycosis cases (75.5%) were classified as mild or moderate infections, with a mean OSI score of 10.25 ± 8.09. This is consistent with previous reports indicating that severe onychomycosis is relatively uncommon [38].

### Etiology of nail fungal infections

A key finding of the study is the distribution of causative fungal pathogens in onychomycosis. Results presented in Table 4 indicate that yeasts were the predominant agents (70.6%), while NDMs and dermatophytes accounted for 29.4% of the cases. For many years, dermatophytes were considered the principal pathogens responsible for onychomycosis [2, 36]. However, recent reports from diverse geographic regions, including Europe [9], Asia [13, 16], Africa [11, 12], and South America [10], indicate a shift toward yeast predominance. The reasons for this shift remain unclear but may involve female gender [38], advanced age [11, 4], frequent exposure to moisture, chemicals, microtraumas [39], and living in hot and humid environments [11], [12, 40].

In this study, the high prevalence of yeast-related onychomycosis likely reflects the characteristics of participants—mainly middle-aged women. Furthermore, this predominance aligns with identified risk factors in the present analysis: nail grooming and water exposure. This highlights the need for accurate pathogen identification to improve epidemiological insight and guide antifungal therapy, given varying drug susceptibilities across the different fungal taxa [20, 21, 41].

In the present study, *Candida* was found to be the most common yeast (77.8% of the yeast isolates), which was consistent with previous reports [9, 14, 15]. The second most frequent yeast was *Trichosporon*, an emerging pathogen that has been reported in up to 50% of toenail onychomycosis cases [42, 43]. The other identified yeast was *Geotrichum*, which has also been documented in other studies [14, 17]. Considering individual species, *Candida albicans* was the leading agent (46.1% of all cases and 83.9% of *Candida* isolates), identical to those from other reports [11, 15, 17, 11]. Rare non-albicans species - *Candida fermentati* (telemorph *Meyerozyma caribbica*), *Candida etchellsii* (*Starmerella etchellsii*), and *Candida cylindracea* - have also been reported as pathogens in diverse patient populations [44, 45]. From a clinical perspective, the leading prevalence of *C. albicans* is significant, as it usually exhibits greater susceptibility to standard antifungal therapies compared with non-albicans *Candida* species [41, 46].

### Filamentous fungi

As shown in Table 4, nearly all recovered filamentous fungi (96.7%) were NDMs. This supports previous evidence that NDMs are emerging pathogens in toenail onychomycosis [15] and may be the dominant cause in some regions [17, 5]. High prevalence of NDMs in mold-associated onychomycosis in this study is concerning, as these infections are often more difficult to treat than those caused by dermatophytes [15, 47].

In the present study, four genera of mold were identified, with *Aspergillus* being the most common (16.7% of all cases and 56.7% of filamentous strains), which is consistent with findings from other studies [47, 49]. The second most frequent mold was *Penicillium* (5.9% of all cases), which is comparable to the rate of 7.2% of positive cases reported in a Canadian study [38]. Presence of *Curvularia* and *Fusarium* in toenail onychomycosis has also been previously reported [50]. Role of *A. flavus* as the leading mold species in this study is concordant with a recent review conducted by Bongomin et al. (2017) [51]. It is noteworthy that dermatophytes were rare in their study. This may be related to the demographics of the participants-middle-aged women, a group with a lower incidence of these fungal infections [42, 53]. There is evidence that certain female hormones, such as progesterone, have the capacity to inhibit the development of dermatophytes [52].

### Relationship between fungal etiology and clinical variables

One of the principal findings of this study was the observed association between fungal etiology and clinical variables. Mold infections were significantly more common in older patients compared to yeast infections. This observation is consistent with other epidemiological studies that show dermatophyte infections are more prevalent in younger adults, while yeast or NDM infections are more frequent in older individuals [11, 4, 38]. Higher frequency of molds observed in older participants in this study may reflect the predominance of NDMs among mold isolates. It is noteworthy that although yeast was the most common cause of infection in this study, it was associated with milder clinical presentations compared to mold infections. This aligns with previous findings by Gupta AK et al. (2024), who noted that yeast infections seem to produce milder clinical manifestations [38]. Similarly, Dubljanin et al. (2021) employed Scoring Clinical Index for Onychomycosis and observed a mean value of 17.4 for dermatophytes, 10.8 for NDMs, and 8.7 for yeasts [37]. Recognition of the link between mild clinical presentation and potential yeast involvement may help guide empirical treatment decisions, a common approach in clinical practice [22]. Findings of the present study suggest that, in mild to moderate cases, antifungal agents with strong anti-yeast activity, such as azoles, may be a more suitable choice than terbinafine, which exhibits variable activity against both yeasts and NDMs [8].

This study had some limitations. First, the diagnosis of onychomycosis relied on direct examination and fungal isolation. While these methods are widely used due to their practicality and cost-effectiveness, their sensitivity may be lower than that of techniques, like Periodic Acid-Schiff staining or PCR, a factor to be considered in future studies [53]. Additionally, confirmatory identification was based on ITS sequencing. Although ITS sequencing is a well-established and validated tool in fungal taxonomy, it may not fully capture the complexity of fungal diversity [54]. However, findings of this study provide valuable insights into toenail onychomycosis, contributing to the prevention and treatment of this difficult-to-treat fungal infection.

## Conclusion

In summary, the results suggest that cosmetic nail procedures and occupational exposure to seafood are risk factors for toenail onychomycosis among female traders in Nghe An. Most infections were mild to moderate and caused by yeast, particularly *Candida* species, while more severe cases were generally associated with non-dermatophyte molds. These findings highlight the need to adapt public health treatment strategies and strengthen disease prevention.
